# Predictors of loss to follow-up among children registered in an HIV prevention mother-to-child transmission cohort study in Pernambuco, Brazil

**DOI:** 10.1186/1471-2458-14-1232

**Published:** 2014-11-27

**Authors:** Pedro Alves da Cruz Gouveia, Gerlane Alves Pontes da Silva, Maria de Fatima Pessoa Militão de Albuquerque

**Affiliations:** Department of Tropical Medicine, Federal University of Pernambuco, Recife, Brazil; Instituto de Medicina Integral Prof. Fernando Figueira (IMIP), Recife, Brazil; Department of Public Health, Aggeu Magalhães Research Center, Recife, Brazil; Programa de Pós-Graduação em Medicina Tropical, Bl. A – Térreo do HC/UFPE, Cidade Universitária, Av. Prof. Moraes Rego s/n, 50670-901 Recife/PE, Brasil

**Keywords:** Lost to Follow-Up, HIV, Infectious Disease Vertical Transmission, Risk Factor, Brazil

## Abstract

**Background:**

Mother-to-child transmission of HIV (MTCT) is the major form of acquiring the disease among children. The loss to follow-up (LTF) of mothers and their children is a problem that affects the effectiveness of programs for the prevention of mother-to-child transmission (PMTCT). The aim of this study is to identify risk factors associated with the LTF of HIV-exposed children in the state of Pernambuco, Brazil.

**Methods:**

A retrospective cohort study was carried out with 1200 HIV-exposed children born between 2000 and 2009, registered up to the age of 2 months in a public health PMTCT program. Children were considered LTF if they did not return for scheduled visits to monitor infection status. Univariate and multivariate logistic regression analyses were conducted to identify risk factors for LTF.

**Results:**

A total of 185 children (15.4%; CI: 95%: 13.4 - 17.4%) met the case definition of LTF before the determination of serological HIV status. Risk factors independently associated with LTF were mother-child pairs who reside in rural and remote areas (OR 1.86; 95% CI: 1.30-2.66) and mothers who use illicit drugs (OR 1.8; 95% CI: 1.08-3.0). Initiation of the PMTCT during pregnancy was a protective factor for LTF (OR 0.69; 95% CI: 0.49-0.96).

**Conclusions:**

The decentralization of support services for HIV-exposed children to other cities in the state seems to be crucial for the accurate monitoring of outcomes. It is also important to introduce additional measures addressing mothers who are drug users so that they remain in the program: an intensive follow-up program that actively searches for absentee mother-child pairs, support from social services and treatment for drug-dependency. The findings of this study highlight the importance of diagnosing mothers as early as possible in order to conduct a more complete follow-up period of the children. Solving the above-mentioned problems is a challenge, which must be overcome so as to improve the quality of PMTCT.

## Background

Mother-to-child transmission (MTCT) of HIV is the major form of acquiring the disease in children. One of the challenges when analyzing the potential effectiveness of interventions brought about by programs for the prevention of mother-to-child transmission (PMTCT) is the loss to follow-up (LTF) of mothers and their children. Poor follow-up of HIV-exposed children remains a major barrier, undermining the ability to support safe infant feeding practices and to measure HIV-free survival at 18 months [1]. Moreover, some of the same variables that affect LTF rates may also contribute to MTCT [2], so it is important to fully understand the determinants of LTF. Achieving worldwide elimination of pediatric HIV infection requires the identification of service integration models that maximize retention in PMTCT programs [3].

In Africa many studies have demonstrated a very high LTF rate of children of between 36.9% and 68.4% [1, 2, 4]. Such high LTF rates experienced by PMTCT programs preclude them from identifying and managing HIV-infected children [5]. This is a reflection of the failings in PMTCT and is one of the reasons that more than 90% of the children who acquired HIV infection in 2011 live in sub-Saharan Africa [6]. In India, 19.6% of women were LTF after delivery and the significant factors associated were poor education, low economic status, and registration after 20 weeks of pregnancy [7]. However, there is a lack of literature on the risk factors for LTF of HIV-exposed children within PMTCT programs in Latin America and Brazil.

Brazil was the first developing country to introduce a national plan for the prevention of MTCT [8], which gained international notoriety due to its commitment in implementing actions that responded to HIV infection [9]. Despite this progression, prevention has not been universally applied on a nationwide basis [10, 11], especially in the Northeast where there is poor health coverage [12]. In the state of Pernambuco, Northeast of Brazil, Gouveia et al. encountered a MTCT rate of 9.2% and an incomplete follow-up rate of 15.4% [13]. These findings have led us to conduct further examination of the issue in this study, with the risk factors associated with LTF as the main outcome. A profile analysis of the children who did not return for follow-up may help to understand the problem and hence provide an insight on how to address it.

## Methods

### Study design

A retrospective cohort study was carried out at the Instituto de Medicina Integral Professor Fernando Figueira (IMIP), the referral center in the state of Pernambuco for pediatric HIV and located in the capital, Recife. IMIP is a teaching hospital that attends patients exclusively from the Brazilian public healthcare system, and PMTCT services are offered free of charge (counseling, HIV-testing, antiretroviral treatment, routine antenatal, natal and postnatal services). The study population consisted of children born from HIV-infected women registered before two months of age on the PMTCT program at IMIP. The follow-up period of this study did not begin from the prenatal period but rather from the child’s first consultation on the PMTCT program. Participants were enrolled from January 2000, when the compulsory registration of pregnant women with HIV became law in Brazil, to December 2009, and children were followed up for at least eighteen months, until June of 2011. Second-born twins were excluded from the study because it was assumed that the factors associated with LTF would be the same for children of multiple births.

Routine services provide monthly postnatal consultations until 12 months of age, and thereafter every two months, until 24 months of age. Postnatal visits include the following care: zidovudine for infants up to the sixth week of life, provision of infant feeding formula and counseling for non-breastfeeding, primary prophylaxis for Pneumocystis jiroveci pneumonia with cotrimoxazole from 6 weeks onwards up to rule out HIV infection, defining the HIV infection status of children, immunization, development and growth monitoring, and antiretroviral provision, if necessary. During the study period, there were no additional measures in routine care to minimize the LTF.

The definition of HIV infection status of children has already been described in the previous study [13]. The children were followed for scheduled monthly visits to determine the HIV serostatus until 18 months of life. Children were only considered LTF if did not return for scheduled visits before determining their serological HIV status. If a child missed subsequent visits after determining HIV status, it was not considered LTF.

### Data collection

The study received approval from the IMIP Research Ethics Committee. Retrospective review of medical records was performed. Basic demographic characteristics of the mother-child pair enrolled in the program included: age, educational level, place of residence, illicit drug use (current use or in the last three months of marijuana, cocaine or crack), timing of HIV diagnosis, initiation moment of PMTCT, year of child birth, prenatal care, prenatal care at IMIP and delivery at IMIP. Data were inserted in an Excel spreadsheet and systematically checked for internal consistency. The final database was converted and analyzed using Stata-11.0.

### Statistical analysis

Univariate analysis was performed in order to identify factors associated with LTF. In order to measure the association between predictors and LTF, the odds ratios (OR) were calculated with a 95% confidence interval (CI), and chi-squared tests or Fischer’s Exact Test were performed. A multivariate logistic regression analysis was performed with the inclusion of independent variables with p < 0.2 in the univariate analysis. In order to identify the model with the best adjustment and biological plausibility, the stepwise forward method was used and all findings had a significance level of 5%.

## Results

A total of 1200 children were registered in the retrospective cohort after the exclusion of eight second-born twins. Of these children, 185 (15.4%; CI: 95%: 13.4 - 17.4%) met the case definition of LTF. During the study period seven children died, but were not excluded or considered LTF because they died in the presence of class B or C diagnosis according to CDC pediatric case definition of HIV-1/AIDS.

A minority of children was born preterm at <37 weeks (12.8% of 1041 registers) with low birthweight, characterized as <2500 g (18% of 1082 registered). Most deliveries were caesarean (67.9%) and maternal breastfeeding occurred in 9.6% of cases. Most children were from the state capital and surrounding metropolitan region, and 24.9% were from rural and remote areas of the state.

The mean age of mothers was 25 (95% CI: 24.7–25.3), ranging from 15 to 45 years. Most women had received only basic education (66.2%) and prenatal care (91.9%). Among the 1083 women for whom information was available regarding illicit drug use, 9.3% reported current or prior use of marijuana, cocaine or crack. Most mothers received the HIV diagnosis before delivery: 39.5% during pregnancy and 37.7% before pregnancy. Maternal diagnosis occurred during delivery in 15.3% of cases and after childbirth in 7.5%.

The PMTCT was started during prenatal care in 65.3% of cases and later during delivery in 14.6%. Prophylactic measures began in only 10% for newborn, and unfortunately 10.1% of children exposed to HIV did not receive any kind of prophylaxis.

Table 1 compares the characteristics of the 1015 mother-child pairs who completed follow-up with the 185 pairs who lost follow-up before determining the child’s HIV status and demonstrates the results of the univariate analysis of various potential factors associated with LTF. Maternal education, age, place of origin, use of illicit drugs, prenatal care at IMIP and the moment of initiating PMTCT were associated with LTF.

**Table 1:** **Univariate analysis of predictors for lost follow-up of HIV-exposed children in IMIP, Brazil, 2000-2009**

	Lost follow-up		Complete follow-up		Odds-ratio		
	N	(%)	N	(%)	(CI 95%)	χ ^2^	p-value
**Maternal age**							
≥ 20 years	153	(14.8%)	880	(85.2%)	1		
< 20 years	30	(21.1%)	112	(78.9%)	1.54 (0.99-2.39)	3.79	0.053
Unknown	2		23				
**Level of education**							
> 8 years of study	49	(12.7%)	338	(87.3%)	1		
≤ 8 years of study	132	(17.4%)	627	(82.6%)	1.45 (1.02-2.07)	4.31	0.039
Unknown	4		50				
**Place of residence**							
Capital or metropolitan region	122	(13.5%)	779	(86.5%)	1		
Rural and remote areas	63	(21.1%)	236	(78.9%)	1.70 (1.22-2.39)	9.76	0.002
**Use of illicit drugs**							
No	149	(15.2%)	833	(84.8%)	1		
Yes	23	(22.8%)	78	(77.2%)	1.65 (1.00-2.71)	3.96	0.049
Unknown	13		104				
**Time of maternal HIV diagnosis**							
Before/during pregnancy	133	(14.8%)	768	(85.2%)	1		
At/after delivery	48	(18.1%)	217	(81.9%)	1.28 (0.89-1.84)	1.75	0.185
Unknown	4		30				
**Initiation of PMTCT during pregnancy**							
No	78	(19.0%)	333	(81.0%)	1		
Yes	104	(13.5%)	667	(86.5%)	0.67 (0.48-0.92)	6.20	0.013
Unknown	3		15				
**Prenatal care**							
No	17	(19.5%)	70	(80.5%)	1		
Yes	143	(14.5%)	845	(85.5%)	0.70 (0.40-1.22)	1.62	0.203
Unknown	25		100				
**Prenatal care at IMIP**							
No	85	(17.4%)	404	(82.6%)	1		
Yes	63	(12.3%)	448	(87.7%)	0.67 (0.47-0.95)	5.06	0.024
Unknown	37		163				
**Delivery at IMIP**							
No	59	(15.0%)	335	(85.0%)	1		
Yes	89	(14.3%)	533	(85.7%)	0.95 (0.66-1.35)	0.08	0.769
Unknown	37		147				
**Year of child birth**							
2005-2009	121	(16.4%)	616	(83.6%)	1		
2000-2004	64	(13.8%)	399	(86.2%)	0.82 (0.59-1.13)	1.47	0.226

IMIP: Instituto de Medicina Integral Professor Fernando Figueira; PMTCT: prevention of mother-to-child transmission.

The results of the final multivariate model conducted to identify the predictors associated with LTF are shown in Table 2. Mother-child pairs who live in rural and remote areas of the state were 1.86 (95% CI: 1.30-2.66) times more likely to be LTF than those from the capital and metropolitan area, after control for other covariates. A mother who uses illicit drugs was 1.8 (95% CI: 1.08-3.0) times more likely to be LTF than a mother who did not. Initiation of PMTCT during pregnancy was a protective factor for LTF (OR 0.69; 95% CI: 0.49-0.96), in relation to initiating PMTCT at delivery or with the newborn and to missed PMTCT opportunities.

**Table 2:** **Final multivariate model of predictors associated with lost follow-up of HIV-exposed children in IMIP, Brazil, 2000-2009**

	Adjusted odds-ratio	Confidence interval 95%	p-value
**Variable**			
**Residence in remote areas**	1.86	1.30-2.66	0.001
**Illicit drugs use**	1.80	1.08-2.99	0.024
**Initiation of PMTCT during pregnancy**	0.69	0.49-0.96	0.029

IMIP: Instituto de Medicina Integral Professor Fernando Figueira; PMTCT: prevention of mother-to-child transmission.

## Discussion

This is the first study carried out in Brazil to identify predictive factors for LTF of HIV-exposed children as a primary outcome. The LTF encountered (15.4%) is similar to other Brazilian studies on PMTCT, where follow-up of children was not the primary outcome. Using a similar case definition, Succi [10] and Matida et al. [14] found that final HIV status was undefined respectively for 18.8% and 14.5% of the children. Studies in the northeastern region indicate slightly higher rates: in Salvador 25% of children born to HIV-infected mothers were LTF [15]; and in one northeastern capital, 31% of mothers did not return to the service and it was therefore not possible to establish the HIV status of their children [16]. When comparing the results from this study, it is important to note that the LTF herein reported include only those children lost after delivery and thus not the loss of mothers from pregnancy to delivery.

One protective factor encountered for LTF was the initiation of PMTCT during pregnancy (OR 0.69; 95% CI: 0.49-0.96), when compared to initiating PMTCT at delivery or with the newborn and to missed PMTCT opportunities. Women who register earlier have the opportunity of being booked in for more medical appointments, and also have more time to deal with their HIV diagnosis, which might result in better follow-up [7]. On the other hand, women with undocumented HIV infection late into pregnancy or during delivery present high-risk behavior and live under extremely stressful social conditions, thus resulting in a significant degree of vulnerability [17]. The Cox regression analysis in a study in South Africa revealed that late antenatal attendance (≥28 weeks gestation) when compared to attendance during the first trimester, was a predictor for LTF in children [1]. Similarly in India, a woman who was registered after 20 weeks of pregnancy was 1.75 times more likely to be LTF than a woman who was registered within the first 20 weeks of pregnancy [7]. These findings indicate that it is important to focus efforts on registering women during early pregnancy, in order to retain them in the program. The LTF of children born to women who initiate PMTCT later emphasizes the need to identify this group of patients for intensive follow-up and educate them on the importance of monitoring both for themselves and for their infants.

A mother-child pair from rural and remote areas of the state was 1.86 times more likely to be LTF than a mother-child pair from the capital and metropolitan area. This may be explained by the fact that in remote areas mothers are forced to travel long distances in order to reach the nearest hospital, which necessarily involves high costs. For many families, the high cost of transport is a major barrier in preventing access to HIV treatment [18]. An African qualitative study identified that transport costs to a centralized PMTCT service constituted a serious burden in rural areas [19]. A qualitative Brazilian study on adherence to prenatal care by HIV-positive women who failed to receive prophylaxis for MTCT supports the encountered data, indicated that problems were caused because of having to travel long distances to the health services as well as the travel costs involved [20]. Furthermore, the unavailability of financial resources for delivering samples to the more remote regions delays diagnosis and contributes to LTF [21]. The problem of LTF associated with an ‘overly centralized’ hospital-based PMTCT implementation strategy is recognized, therefore improving geographical access by decentralizing PMTCT to peripheral sites needs to become a major priority [22]. In the state of Pernambuco, a transition is occurring from a centralized to a decentralized program. However, 25% of children attended at IMIP arrive from the interior of the state, thus revealing failings in the health system outside the capital. Strong efforts are required to train teams in other cities and towns throughout the state, which should be chosen on the basis of population and geographical access. Adopting a strategy of service integration models could help maximize retention in PMTCT programs.

It is well known that in adult patients living with HIV the use of illicit drugs is an independent risk factor for LTF and injecting drug users have a higher incidence of LTF [23, 24]. Among pregnant women, prenatal illicit drug use is associated with non-adherence to antiretroviral treatment [25, 26]. Drug use can affect the psychopathological conditions for patient adherence to treatment and to maintaining follow-up, so it is considered difficult to register and retain this population in care [23]. Furthermore, illicit drug use can also exacerbate situational barriers, such as financial problems, brought about by unemployment [25]. In Brazil, there is no significant difference between genders in the use of alcohol, tobacco, and illicit drugs [27]. Moreover drug abuse among females of reproductive age is a problem particularly in younger females, because it increases the risk of teenage pregnancy [28]. The present study has demonstrated that women reporting a history of illicit drug use have 1.8 times more risk of LTF and supports the imminent need for effective strategies to improve adherence in this population during pregnancy and after delivery. Satisfying the health needs of such a marginalized group requires much more than the “usual care”: intensive approaches are needed to provide social support, substance abuse treatment and dignified care in order to facilitate the improved continuity of attendance for these women and children [17].

One reason cited by mothers for LTF is the wellbeing of their children [4]. Studies suggest that in the absence of infant illness, mothers do not perceive the need to seek medical care and have stressed the need to improve the quality of information provided to mothers on the importance of follow-up [29]. After 6 weeks of follow-up, given that pharmacological intervention has terminated, it is likely that the perception of mothers regarding the advantages of attending follow-up decreases [30]. The majority of babies may be in good health and the fear of a possible positive result for the baby may prevent the women from bringing them in for testing [30]. They may move house without informing the services. Another reason might be that when the infants’ first PCR/DNA HIV test result (at 45 days of age) is negative, mothers may decide that it is not necessary to continue with further follow-up [31]. In this study, children’s health was not analyzed, but a comparison of the 1015 mother-child pairs who completed follow-up with the 185 pairs who were LTF showed similar main risk factors associated with HIV transmission [13]. This data suggests that children who LTF are healthy and had no increased risk of being infected.

The present study has the limitation of having been conducted at a single center, and the study population may not have been representative of children exposed to MTCT throughout the State of Pernambuco. However, this may be an offset, as the chosen center, IMIP, receives the greatest regional demand for HIV-exposed children. During the study period, IMIP attended around 60% of all infected children aged five years and under from the entire state. One limitation of the PMTCT program was that patients were not contacted (either by letter, telephone or home visit) when they missed their scheduled appointments with the doctor. A study in Haiti indicated that women may appear to be LTF when, in fact, they might simply be attending a different clinic [32]. Hence, contacting patients could indicate the reasons for not returning for care and therefore exclude children attending follow-up in other services.

## Conclusions

The LTF of children is a great problem for evaluating the effectiveness of prophylaxis to prevent perinatal HIV transmission. In Pernambuco, the LTF is 15.4% and is associated with mothers from rural and remote areas of the state and the use of illicit drugs. The decentralization of support services for HIV-exposed children to other cities or towns in the state is crucial for the accurate monitoring of outcomes. It is also important to introduce additional measures so that mothers who are drug users remain in the program: intensive follow-up with an active search for absentee mother-child pairs, support from social services and drug dependency treatment. The findings of this study highlight the importance of diagnosing mothers as early as possible in order to conduct a more complete follow-up period of the children. Solving the above-mentioned problems is a challenge, which must be overcome so as to improve the quality of PMTCT.

## Abbreviations

IMIP: Instituto de Medicina Integral Professor Fernando Figueira
LTF: Loss to follow-up
MTCT: Mother-to-child transmission of HIV
PMTCT: Prevention of mother-to-child transmission.
